# miR-30c-1 encourages human corneal endothelial cells to regenerate through ameliorating senescence

**DOI:** 10.18632/aging.202719

**Published:** 2021-03-19

**Authors:** Younghwan Bae, Jin Sun Hwang, Young Joo Shin

**Affiliations:** 1 Department of Ophthalmology, Hallym University Medical Center, Hallym University College of Medicine, Seoul, Republic of Korea

**Keywords:** miR-30c-1, human corneal endothelial cells, senescence, proliferation, TGF-β

## Abstract

In the present study, we studied the role of microRNA-30c-1 (miR-30c-1) on transforming growth factor beta1 (TGF-β1)-induced senescence of hCECs. hCECs were transfected by miR-30c-1 and treated with TGF-β1 to assess the inhibitory effect of miR-30c-1 on TGF-β1-induced senescence. Cell viability and proliferation rate in miR-30c-1-transfected cells was elevated compared with control. Cell cycle analysis revealed that cell abundance in S phase was elevated in miR-30c-1-treated cells compared with control. TGF-β1 increased the senescence of hCECs; however, this was ameliorated by miR-30c-1. TGF-β1 increased the size of hCECs, the ratio of senescence-associated beta-galactosidase-stained cells, secretion of senescence-associated secretory phenotype factors, the oxidative stress, and arrested the cell cycle, all of which were ameliorated by miR-30c-1 treatment. miR-30c-1 also suppressed a TGF-β1-induced depolarization of mitochondrial membrane potential and a TGF-β1 stimulated increase in levels of cleaved poly (ADP-ribose) polymerase (PARP), cleaved caspase 3, and microtubule-associated proteins 1A/1B light chain 3B II. In conclusion, miR-30c-1 promoted the proliferation of hCECs through ameliorating the TGF- β1-induced senescence of hCECs and reducing cell death of hCECs. Thus, miR-30c-1 may be a therapeutic target for hCECs regeneration.

## INTRODUCTION

Human corneal endothelial cells (hCECs) are characterized as playing a pivotal role in making the cornea transparent and as not regenerating *in vivo* [1]. In response to injury, hCECs undergo compensatory cellular hypertrophy, which is characterized by increased size and migration of adjacent cells [2, 3]. If the enlarged hCECs no longer compensate the function [2], it results in the bullous, and painful corneal edema [4]. Proliferation of hCECs is blocked *in vivo*, and therefore, there is no alternative to transplantation in hCEC-related disease. Understanding the mechanism that blocks the proliferation of hCECs and temporarily resolving this may be valuable for regenerative treatment of hCEC disease. Cellular senescence is accompanied by a loss of proliferation capacity, cell cycle cessation, and cellular hypertrophy. These changes are similar to changes in hCEC seen *in vivo* during the wound healing process. Thus, inhibition of senescence may be a key mechanism for regeneration of hCECs.

MicroRNA (miRNA) is a short non-coding RNA molecule that is involved in gene expression [5]. It is single-stranded and composed of ~20–25 nucleotides in length [5]. The RNase III enzyme Dicer cleaves the pre-miRNA hairpin to miRNA-5p and miRNA-3p [6]. The 5p strand is located in the forward, and the 3p strand in the reverse [6]. The key function of miRNAs degrades or inhibits messenger RNAs (mRNAs) translation through binding to the complementary mRNAs. miR-30c has been found in murine corneal endothelial cells and is decreased in expression in Fuchs’ corneal endothelial dystrophy (FECD) [7]. The miR-30c family includes miR-30c-1 and miR-30c-2, and their functions may be differ due to sequence differences of miRNA-3p. The role of miR-30c-1 has been controversial, although miR-30c-1 has been suggested to promotes cell cycle progression [8, 9]. while regulation of miR-30c-1 may induce regeneration of hCECs.

Thus, in the present study, we investigated the regulatory role of miR-30c-1 on hCEC regeneration and on TGF-β1-induced senescence of hCECs.

## RESULTS

### miR-30c-1 promotes the proliferation of hCECs

#### Cell viability and proliferation

**Figure 1:**
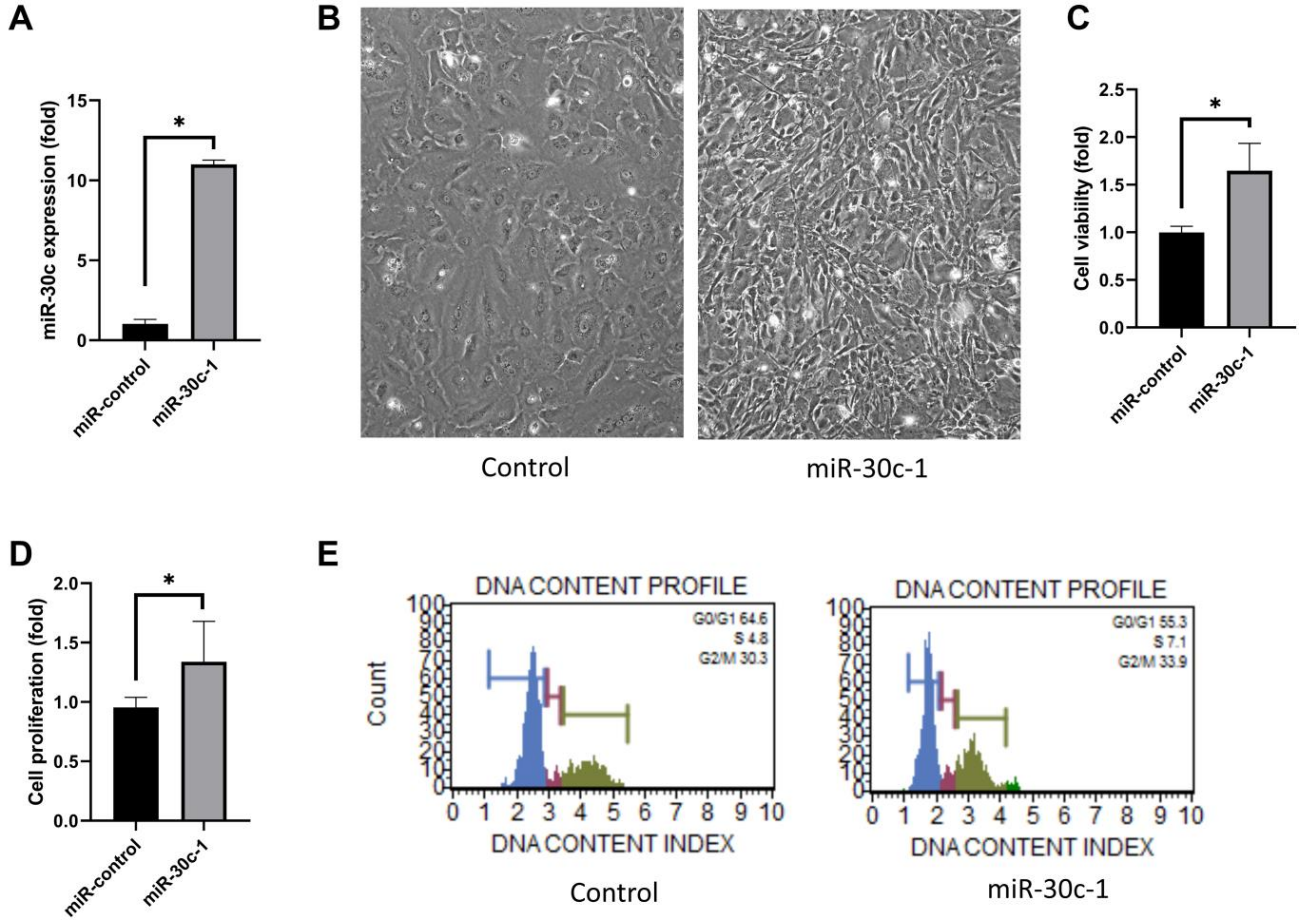
**Cell proliferation induced by miR-30c-1.** (**A**) miR-30c level was elevated in miR-30c-1-treated cells compared with miR-control. (**B**) Representative images of cell shape in control and in miR-30c-1-treated cells. (**C**) Cell viability in control and in miR-30c-1-treated cells measured by CCK-8 assay. (**D**) Cell proliferation in control and in miR-30c-1-treated cells measured by BrdU proliferation assay. (**E**) Cell cycle analysis showing that miR-30c-1 elevated the percentage of cells with S-phase. ^*^statistically significant.

miR-30c level was elevated in miR-30c-1-treated cells compared with miR-control (*p* < 0.001, Figure 1A). hCEC size decreased with miR-30c-1-treatment compared with control (*p* < 0.001, Figure 1B). Cell viability and proliferation rate were elevated in miR-30c-1-treated cells compared with miR-control (*p* < 0.001 and 0.040; Figure 1C–1D). The cellular abundance in S-phase of cell cycle was higher in miR-30c-1-treated cells compared with miR-control (*p* = 0.001; Figure 1E).

#### RNA sequencing

**Figure 2:**
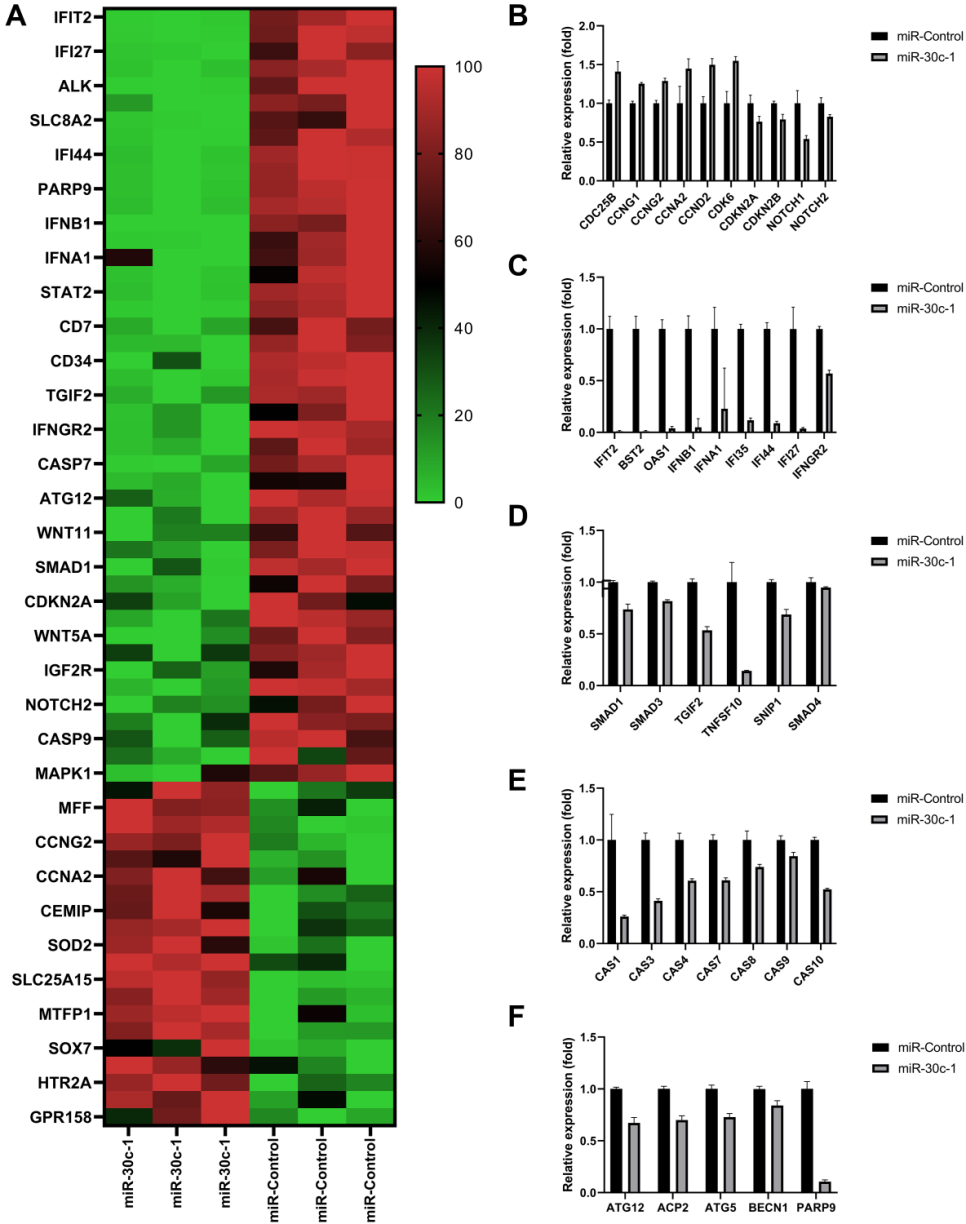
**Results of RNA-sequencing data.** (**A**) Heat map of the relative expression of differentially expressed genes. Comparison of relative expressions of proliferation-associated genes (**B**), interferon (IFN)-associated genes (**C**), transforming growth factor (TGF; (**D**)) caspases (**E**) and autophagy (**F**) between miR-control group and miR-30c-1 group. ^*^statistically significant.

Normalized read counts and fold changes are shown in Figure 2A and Table 1. Relative expressions of cell proliferation-associated genes were elevated and relative expressions of inhibitor genes were reduced in miR-30c-1-treated cells compared with miR-control (*p* < 0.05 for all, Figure 2B, Table 1). Interferon (IFN)-associated genes (Figure 2C), TGF-β-associated genes (Figure 2D), caspases genes (Figure 2E) and autophagy-associated genes (Figure 2F) were decreased in miR-30c-1-treated cells compared with miR-control (*p* < 0.05 for all).

**Table 1:** Specific fold change (FC) values in differentially expressed genes.

**Gene symbol**	**Fold Change (miR-30c-1/miR-control)**	**Log2(FC)**	***p*-value**
**IFIT2**	0.0099863	–6.6458342	0.0001552
**BST2**	0.0174225	–5.8429032	0.0002403
**IFI27**	0.0365895	–4.7724258	0.0013726
**OAS1**	0.0395716	–4.6593894	5.41E-05
**ALK**	0.0468338	–4.4163052	0.0004983
**IFNB1**	0.0488735	–4.3548048	0.0004183
**SLC8A2**	0.0612903	–4.0281969	0.0025017
**HLA-F**	0.0840182	–3.5731542	0.000448
**IFI44**	0.0879615	–3.5069838	1.523E-05
**STAT1**	0.0918394	–3.4447428	3.58E-05
**PARP9**	0.1054564	–3.245281	2.907E-05
**IFI35**	0.1171023	–3.0941583	7.448E-06
**IFNB1**	0.1278718	–2.9672301	0.0001676
**TNFSF10**	0.1417435	–2.8186455	0.0014769
**IFNA1**	0.2279793	–2.1330254	0.0397558
**CASP1**	0.260129	–1.9427008	0.0065726
**STAT2**	0.3039028	–1.7183184	1.162E-05
**IFI16**	0.3461232	–1.5306425	3.402E-05
**CD7**	0.3552655	–1.4930304	0.0017694
**CASP3**	0.4122607	–1.2783713	0.0001307
**CD34**	0.4330144	–1.2075132	0.0011659
**CASP10**	0.5226783	–0.936005	6.75E-06
**TGIF2**	0.5345843	–0.9035108	7.346E-05
**NOTCH1**	0.5389247	–0.8918443	0.0094308
**IFNGR2**	0.5688129	–0.8139739	5.281E-05
**CASP4**	0.6067611	–0.7207996	0.0005647
**CASP7**	0.6100666	–0.7129612	0.0002713
**CIP2A**	0.620878	–0.6876182	0.0123176
**ATG12**	0.6714314	–0.574688	0.000519
**ACP2**	0.6991534	–0.516319	0.0004251
**WNT11**	0.7069583	–0.5003031	0.0078715
**ATG5**	0.7276916	–0.4586009	0.0007922
**SMAD1**	0.737237	–0.4397997	0.0010524
**CASP8**	0.7388248	–0.4366958	0.0077211
**CDKN2A**	0.7620468	–0.3920485	0.0317108
**WNT5B**	0.7692308	–0.3785116	0.000321
**WNT5A**	0.7734093	–0.370696	0.0008211
**CDKN2B**	0.7921014	–0.336243	0.0068403
**IGF2R**	0.8032468	–0.3160849	0.0092538
**SMAD3**	0.8175286	–0.2906589	4.86E-05
**NOTCH2**	0.8281981	–0.2719522	0.018508
**BECN1**	0.8411694	–0.2495317	0.006372
**CASP9**	0.8435622	–0.2454336	0.0077383
**PDGFA**	0.8585895	–0.2199595	0.0460734
**MAPK1**	0.893772	–0.1620212	0.0311704
**COL8A1**	1.0841073	0.1165076	0.0492557
**SIRT1**	1.1383886	0.1869932	0.0403678
**MFF**	1.200156	0.2632219	0.0073113
**CCNG1**	1.2523403	0.3246267	0.0001706
**COL8A2**	1.2644785	0.3385425	0.0206073
**CCNG2**	1.2872408	0.364282	0.0006619
**CDC25B**	1.4089818	0.494653	0.0064732
**CCNA2**	1.4467974	0.5328629	0.0382018
**CCND2**	1.4973629	0.5824239	0.0018972
**CEMIP**	1.502362	0.5872325	0.0174476
**CDK6**	1.5484987	0.6308702	0.0041804
**COL4A4**	1.5797686	0.6597133	0.0199125
**SOD2**	1.7181873	0.7808874	0.0058297
**SLC4A11**	1.7909408	0.8407176	0.0041401
**SLC25A15**	1.8435085	0.8824541	2.931E-05
**CEMIP2**	1.8807528	0.9113102	0.000163
**MTFP1**	1.8954822	0.9225649	0.0119586
**FOXO3**	1.9359241	0.9530224	0.0002565
**COL17A1**	2.0654762	1.0464744	0.0044258
**SOX7**	2.1077586	1.0757097	0.0342846
**AQP7**	2.2103896	1.1443007	0.0256781
**HTR2A**	2.3896723	1.2568128	0.0018409
**GPR20**	4.5950413	2.2000778	0.0123495
**GPR158**	8.34	3.0600474	0.0244592
**COL5A3**	16.64	4.0565835	0.0360859

#### Mitochondrial functions

**Figure 3:**
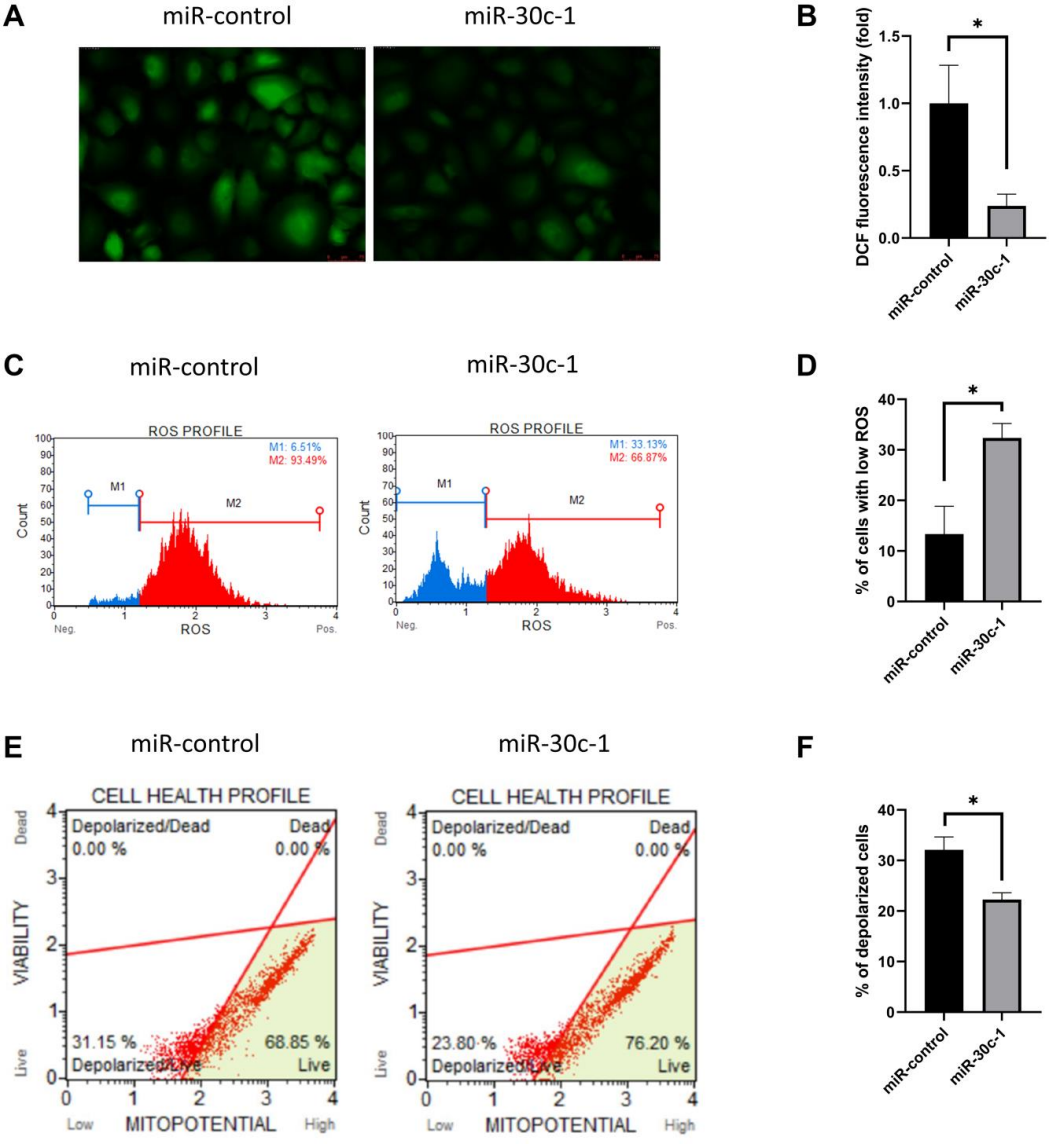
**Oxidative stress level and mitochondrial membrane potential changed by miR-30c-1.** (**A**) Representative images of dichlorofluorescin diacetate staining in control and miR-30c-1-treated cells. (**B**) DCF fluorescence intensity in control and miR-30c-1-treated cells. (**C–D**) Fluorescence intensity of MitoSOX probe was measured by Muse cell analyzer. (**E–F**) Mitochondrial membrane potential was measured using Muse^®^ MitoPotential kit.

DCF fluorescence intensity decreased in miR-30c-1-treated cells compared with miR-control (*p* < 0.001; Figure 3A–3B). Intracellular oxidative stress level measured by Muse analyzer decreased in miR-30c-1-treated cells compared with miR-control (*p* = 0.006; Figure 3C–3D). The mitochondrial membrane potential increased in miR-30c-1-treated cells compared with miR-control (*p* < 0.001; Figure 3E–3F).

### miR-30c-1 ameliorates TGF-β1-induced senescence of hCECs

**Figure 4:**
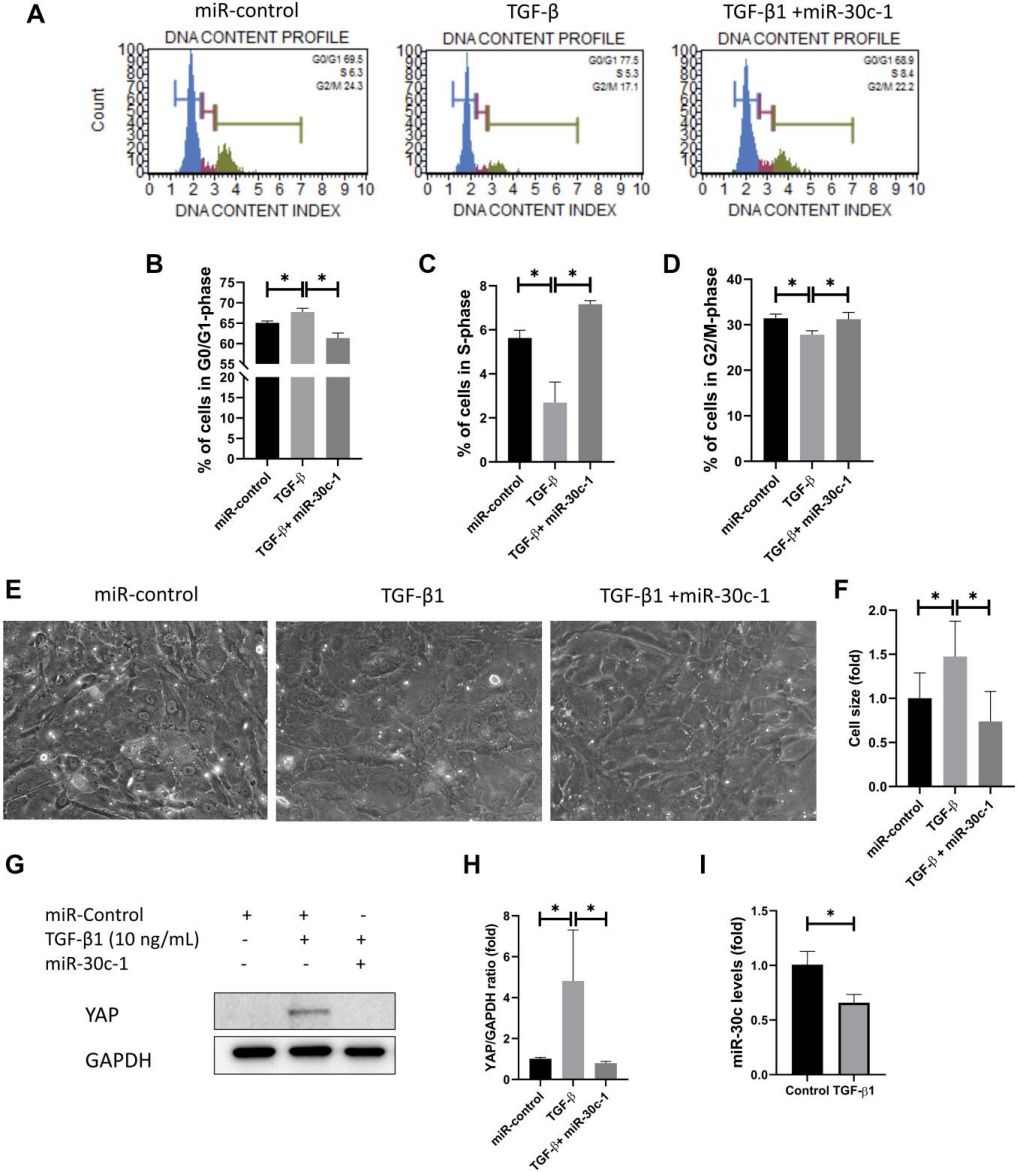
**miR-30c-1 ameliorates TGF-β1-induced cell cycle arrest.** (**A**) Cell cycle analysis was analyzed using DNA content. (**B**) The percentage of cells in S-phase. (**C**) The percentage of cells in G0/G1 phase. (**D**) The percentage of cells in G2/M phase. (**E**) Representative images of cell shape. (**F**) Cell size was increased by TGF-β1 treatment, which was ameliorated by miR-30c-1. (**G**) Representative images of yes-associated protein 1 (YAP). (**H**) YAP levels were quantified. (**I**) miR-30c-l levels were reduced in treatment with TGF-β1. ^*^statistically significant.

Cell cycle analysis was conducted using quantitation of DNA content (Figure 4A). Cell cycle analysis presented that the cellular abundance in G0/G1 phase was elevated following TGF-β1 treatment, but it was not elevated with miR-30c-1 treatment (*p* = 0.010; Figure 4B). The cellular abundance in S-phase and in G2/M-phase was reduced with TGF-β1 treatment compared with miR-30c-1 treatment (*p* = 0.001; Figure 4C–4D). TGF-β1 stimulation also significantly increased cell size (*p* = 0.017) compared with miR-30c-1 treatment (*p* = 0.001; Figure 4E–4F). TGF-β1 caused an elevation in yes-associated protein (YAP) levels compared with miR-30c-1 treatment (*p* = 0.041 and *p* = 0.033, respectively; Figure 4G–4H). miR-30c-1 levels were reduced in treatment with TGF-β1 (*p* = 0.041, Figure 4I).

**Figure 5:**
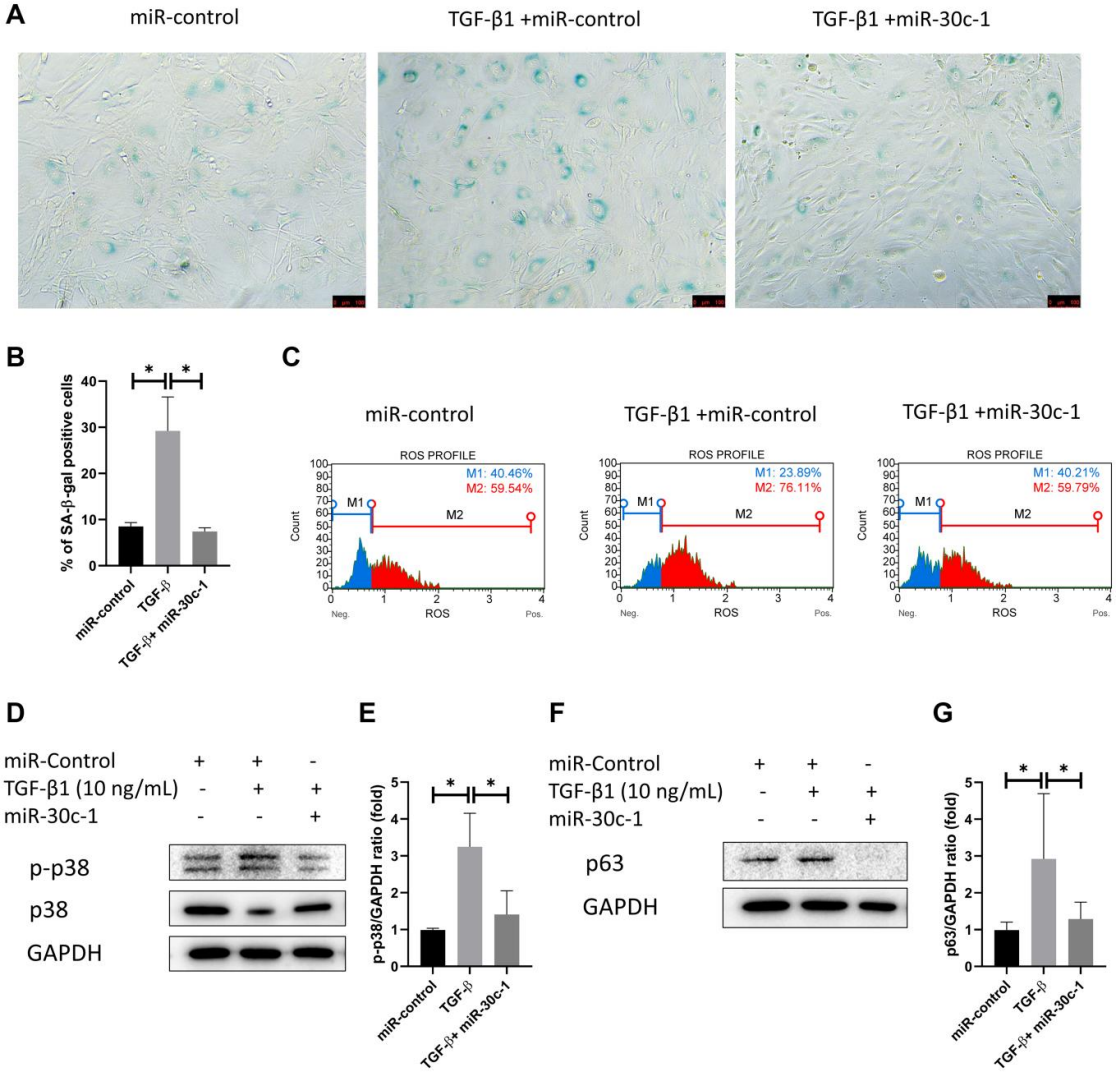
**miR-30c-1 ameliorates TGF-β1-induced senescence.** (**A**) Representative images of senescence-β-galactosidase (SA-β-gal) staining. (**B**) The percentage of SA-β-gal positive cells was quantified. (**C**) Intracellular oxidative stress levels measured by MitoSOX probe. (**D**) Representative images of p-p38 and p38. (**E**) Activation of p38 was quantified. (**F**) Representative images of p63. (**G**) p63 level was quantified. ^*^statistically significant.

The ratio of SA-β-gal-stained cells was elevated with TGF-β1 treatment (*p* = 0.008), which did not occur when miR-30c-1 was present (*p* = 0.007; Figure 5A–5B). Intracellular oxidative stress was increased following TGF-β1 treatment (*p* = 0.035) but was ameliorated by miR-30c-1 (*p* = 0.002; Figure 5C). Additionally, TGF-β1 treatment also increased the levels of p-p38 and p63 (*p* = 0.013 and *p* = 0.019, respectively), but these did not increase when miR-30c-1 was present (*p* = 0.047 and *p* = 0.043, respectively; Figure 5D–5G).

**Figure 6:**
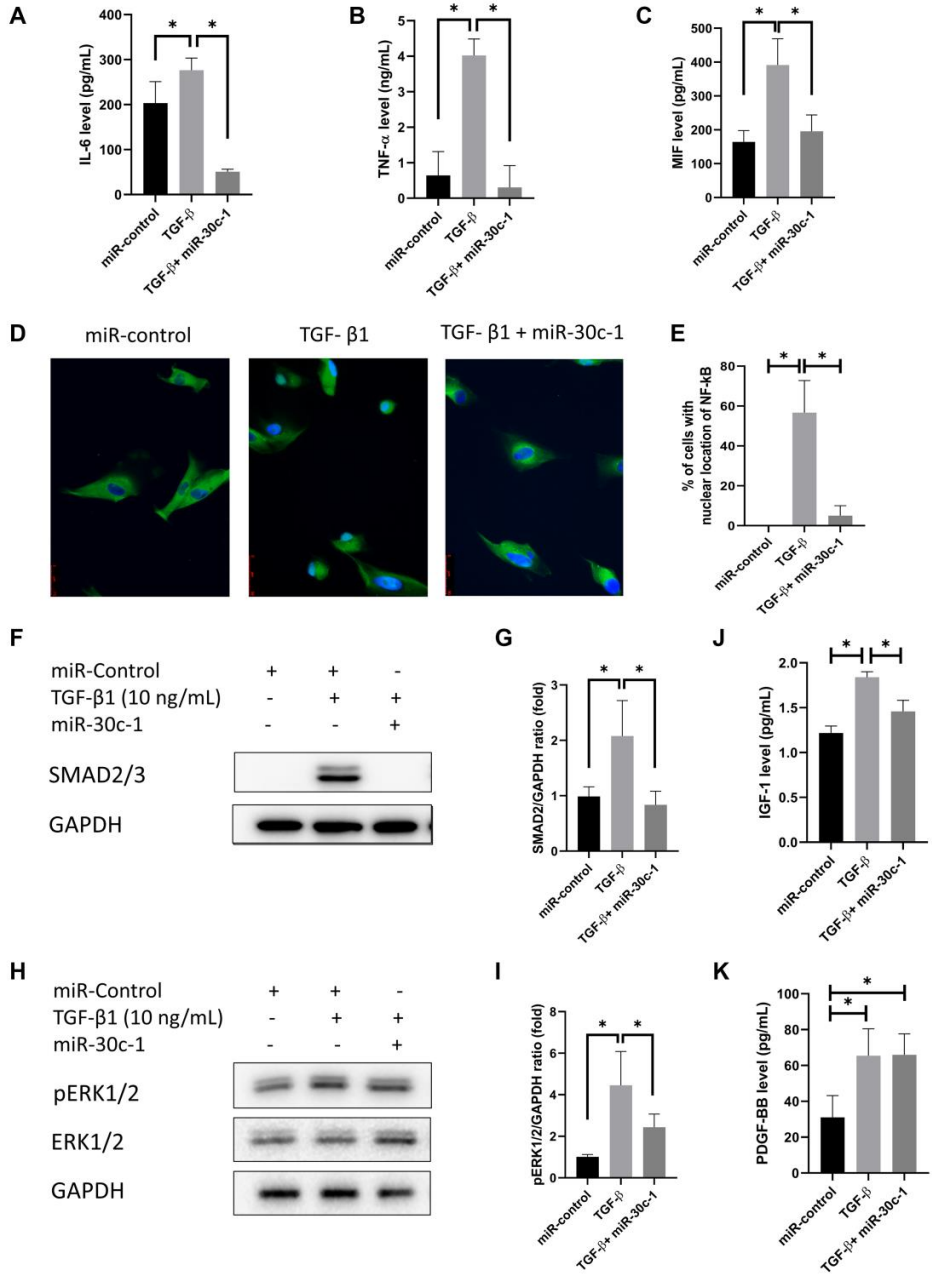
**Senescence-associated secretory phenotype (SASP) factors.** (**A–C**) Interleukin-6 (IL-6; A), tumor necrosis factor-α (TNF-α; B), macrophage migration inhibitory factor (MIF; C) levels were evaluated by ELISA. (**D**) Representative images of immunofluorescence staining of nuclear factor kappa-light-chain-enhancer of activated B cells (NF-κB). (**E**) Nuclear translocation of Nf-κB was evaluated and quantified. Immunofluorescence staining of NF-κB p65 (green) and nuclear with Hoechst 33342 (blue) was performed. (**F–G**) SMAD2/3 levels were evaluated by western blotting. (**H–I**) pERK1/2 levels were evaluated by western blotting. (**J**) Insulin-like growth factor-1 (IGF-1) levels were evaluated by ELISA. (**K**) Platelet-derived growth factor-BB (PDGF-BB) were evaluated by ELISA. ^*^statistically significant.

Evaluation of SASPs in conditioned medium demonstrated that the levels of IL-6, TNF-α, and MIF increased after TGF-β1 treatment (*p* = 0.030, *p* < 0.001, and *p* < 0.010, respectively), which did not occur following miR-30c-1 treatment (*p* = 0.005, *p* < 0.001, and *p* = 0.021, respectively; Figure 6A–6C). The percentage of NF-κB located in the nucleus was elevated by TGF-β1 but was ameliorated by miR-30c-1 (*p* = 0.001 and *p* = 0.002, respectively; Figure 6D–6E). TGF-β1 also elevated the levels of pERK1/2 and SMAD2/3 (*p* = 0.003 and *p* = 0.012, respectively) but not when miR-30c-1 was present (*p* = 0.048 and *p* = 0.013; Figure 6F–6I). Additionally, IGF-1 and PDGF-BB levels were also increased with TGF-β1 treatment (*p* = 0.001 and *p* = 0.032, respectively) but not in the presence of miR-30c-1 (*p* = 0.183 and 0.044; Figure 6J–6K).

### miR-30c-1 ameliorates TGF-β1-induced cell death of hCECs

**Figure 7:**
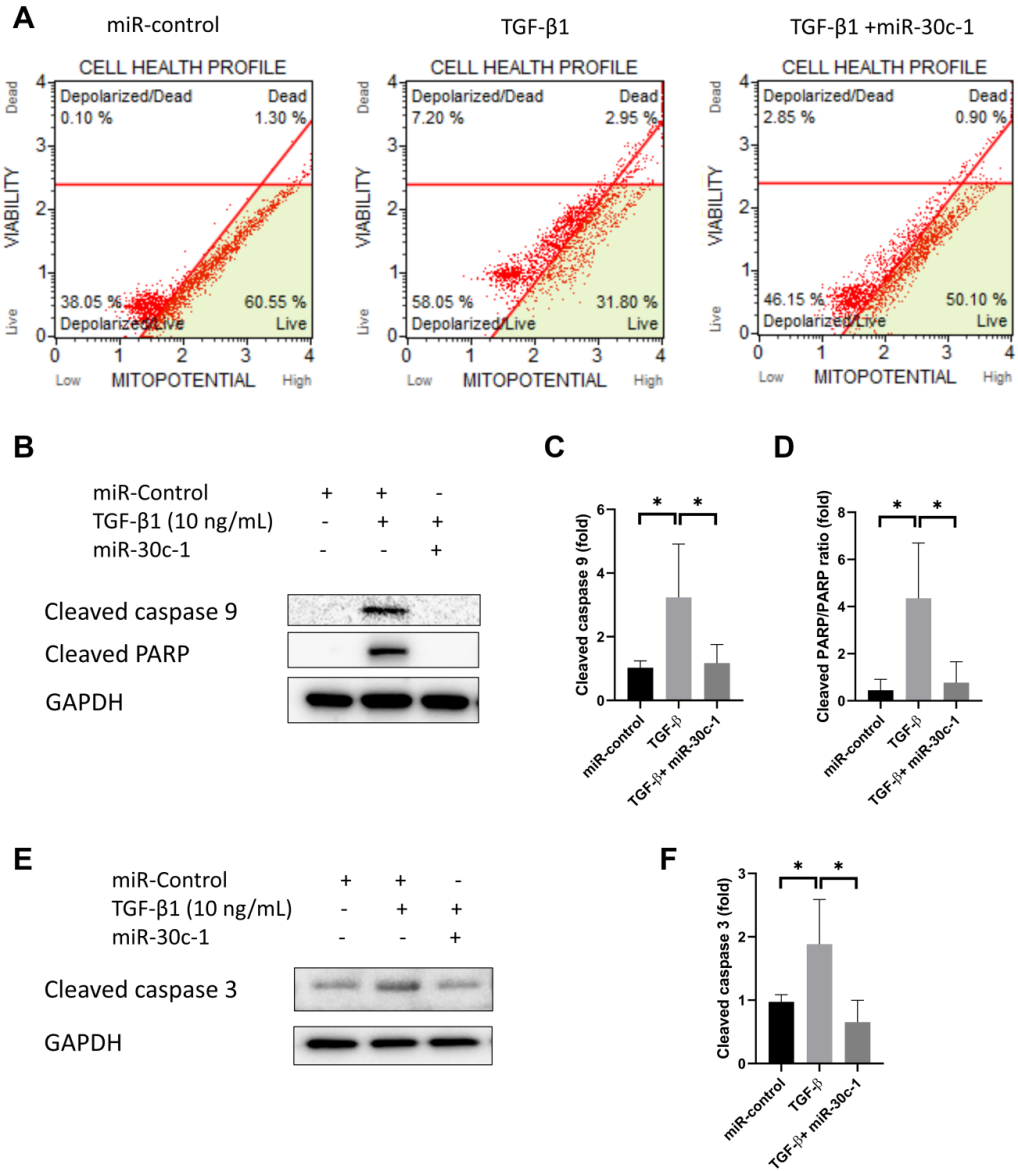
**Cell death by TGF-β1 or miR-30c-1.** (**A**) Mitochondrial membrane potential was measured by MitoPotential kit. (**B–D**) Cleaved caspase 9 and cleaved poly ADP ribose polymerase (PARP) levels were evaluated by western blotting and quantified. (**E–F**) Cleaved caspase 3 was evaluated by western blotting and quantified. ^*^statistically significant.

TGF-β1 treatment promoted the depolarization of mitochondrial membrane potential (*p* = 0.014) but this effect was eliminated with miR-30c-1 treatment (*p* = 0.003, Figure 7A). In addition, TGF-β1 elevated the levels of cleaved caspase 9, cleaved PARP, and cleaved caspase 3 (*p* = 0.034, *p* = 0.012, and *p* = 0.049, respectively), but the presence of miR-30c-1 ameliorated this effect (*p* = 0.047, *p* = 0.019, and *p* = 0.011, respectively; Figure 7B–7F).

**Figure 8:**
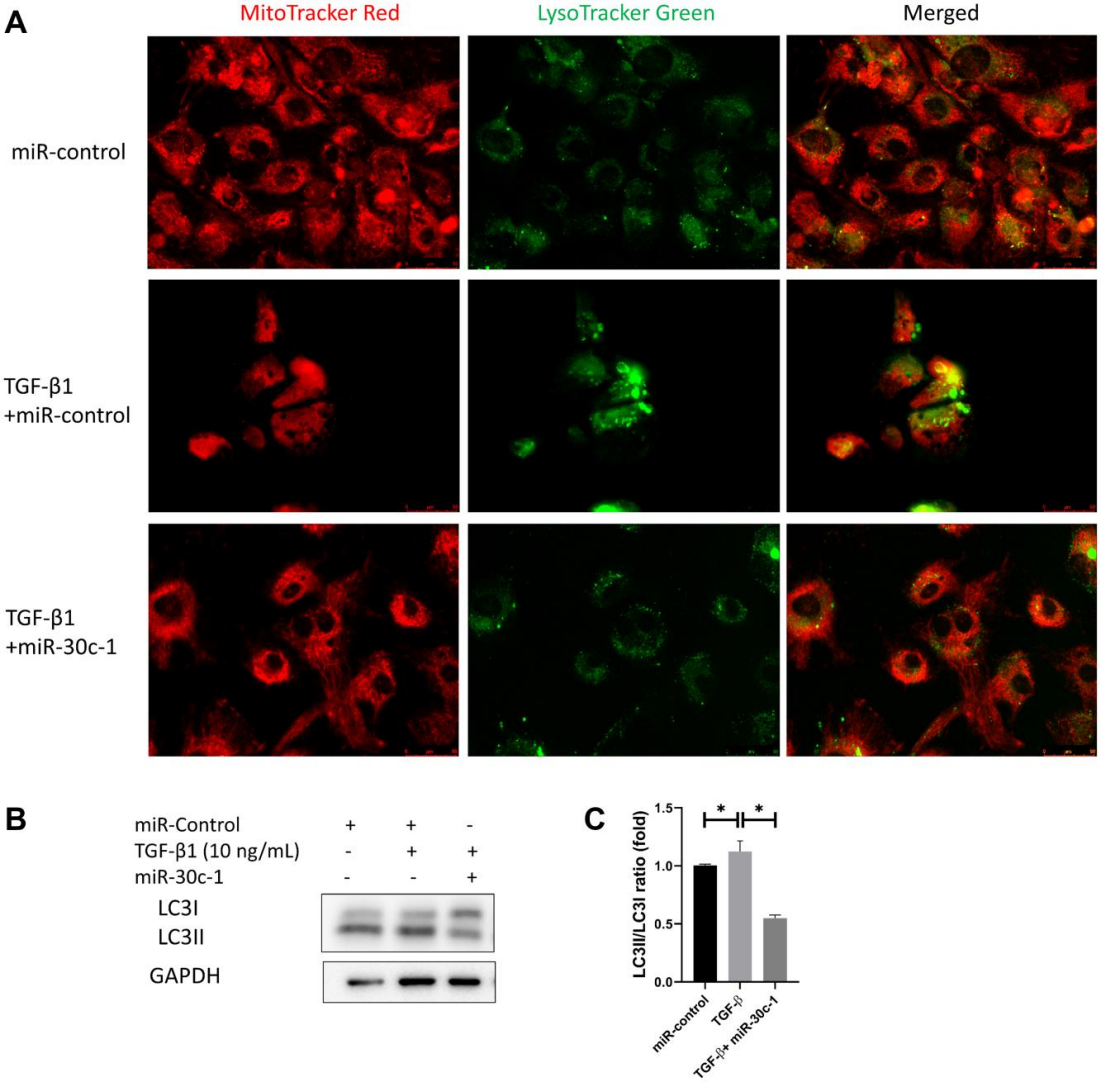
**Autophagy by TGF-β1 or miR-30c-1.** (**A**) Mitochondria (red) and lysosomes (green) were staining. (**B**) Representative images of microtubule-associated protein 1A/1B-light chain 3 (LC3). (**C**) LC3I and LC3II levels were quantified. ^*^statistically significant.

Lysosomes (green stain) became enlarged and prominent following TGF-β1 treatment, while this effect was removed with miR-30c-1 treatment (Figure 8A). The level of LC3II also increased after TGF-β1 treatment (*p* < 0.001) but not when miR-30c-1 was present (*p* < 0.001; Figure 8B–8C).

## DISCUSSION

### miR-30c-1 promotes the proliferation of hCECs

miR-30c-1 is a component of the miR-30 family and there are six miR-30 genes present in the human genome; [10] the miR-30c-1 gene is positioned at chromosome 1 and the miR-30c-2 gene at chromosome 6 [11], but their roles may differ due to their differing sequences. miR-30c-1 is cut into miR-30c-1-5p and miR-30c-1-3p which then bind to target sites including TGFβR2, [12] p16INK4A, [13] NOV/CCN3, [14] RUNX2, [15] KRAS, [8] SNAI1, [16] SNAI2, TWF1, and VIM [17]. This study revealed that miR-30c-1 promotes cell viability and proliferation in hCECs, which was associated with cell cycle analysis. Proliferation-associated genes such as *CDC25B, CCNG1, CCNG2, CCNA2, CCND2, CDK6, CDKN2A/2B and NOTCH1/2* mRNA expressions were changed in treatment with miR-30c-1. miR-30c-1 has been reported to increase stem cell proliferation. [9, 18, 19] Cyclins drives the cell cycle, [20] and elevated the cyclins levels, which is related with a high proliferation rate [20]. The p16INK4A/CDKN2A protein inhibits CDK4/6-cyclin D and phosphorylation of the Rb protein, thereby inducing cell cycle cessation in G1-phase. miR-30 inhibits p16INK4A/CDKN2A in murine cancer model [13]. Notch1 mediates local cell-cell communication, inhibits cell proliferation, and causes premature cell cycle exit; [21] notch1 can also inhibit Rb phosphorylation in primary endothelial cell and induce cell cycle arrest [22]. miR-30c-1 has been reported to suppress NOTCH signaling [23]. IFN-associated genes and TGF-β-associated genes were decreased with in treatment with miR-30c-1. IFN is a group of signaling proteins that participates in immune reaction against infections and that induces apoptosis [24, 25]. TGF-β regulates various cell functions including fibrosis, senescence and apoptosis [26–28]. miR-30c has been reported to suppress the IFN response by targeting JAK1 and TGF-β response by targeting serpine1 [12, 29, 30]. Caspases and autophagy-associated genes were decreased with in treatment with miR-30c-1. Caspases are the executioners of apoptosis that involved in mediating cell death [31]. Autophagy is a recycling and degradative process that delivers cytoplasmic materials to the lysosome [32] and associated with cell death [33].

In the present study, we found that miR-30c-1 reduced intracellular oxidative stress and increased mitochondrial membrane potential. Oxidative stress participates in pathogenesis of FECD or hCEC diseases. Reactive oxygen species (ROS) are produced in mitochondria and excess ROS leads to disruption of cellular function, senescence, inflammation, and apoptosis [34]. miR-30c-5p has been described to suppress oxidative stress-caused cardiomyocyte apoptosis and p53 expression [35]. Mitochondrial membrane potential is a powerful regulator of mitochondrial generation of ROS that performs physiological and pathological functions and is a component of the quality control machinery of mitochondria [20]. miR-30c mimics have been shown to significantly preserve mitochondria membrane potential [36]. In conclusion, miR-30c-1 increased cell viability and proliferation, modulated signaling pathways, and reduced oxidative stress. Thus, miR-30c-1 may be a target for regeneration of hCECs.

### miR-30c-1 ameliorates TGF-β1-induced senescence of hCECs

This study showed that TGF-β1 induces senescence in hCECs. Senescence is cell cycle cessation and is accompanied by cell shape changes, metabolic reprogramming, and release of the SASP [20]. Staining of SA-β-gal is widely used as a sign for senescence [37]. In this study, the ratio of SA-β-gal-stained cells elevated after TGF-β1 treatment, while other senescence-associated changes including cell enlargement, increase of ROS levels and depolarization of mitochondrial membrane potential occur after TGF-β1 treatment. TGF-β1 inhibited cell proliferation and induced cell hypertrophy, which is similar to the wound healing process with *in vivo* hCECs. TGF-β1 blocks cell cycle progression as it prevents cyclin D production and increases INK4, which inhibits RB phosphorylation through binding to CDK4/6 and causing the redistribution of p27 to CDK2-cyclin E. TGF-β1 also increases intracellular oxidative stress level by inducing NADPH oxidases and suppressing antioxidant systems, which results in redox imbalance [38]. Excess ROS leads to apoptosis through the loss of mitochondrial membrane potential [39], which enhances apoptosis by secretion of apoptogenic factors and decline of energy production [20].

In this study, TGF-β1 induced senescence and increased intracellular oxidative stress levels, which were ameliorated by treatment with miR-30c-1. During senescence, TGF-β1 stimulus increased intracellular oxidative stress level [40], which activates p38 [18] and, subsequently, the p53 growth arrest pathway [18]. Cell cycle arrest is linked to accumulated SA-β-gal, while p63 has been known as a mediator of senescence and aging [41]. DNA damage by ROS activates p63 regulation of AMPK and sirtuin 1 (SIRT1) activity, and induces mitochondria dysfunction. TGFβR2 is a validated target of miR-30c-1-3p [12], which binds to the 3'-UTR of TGFβR2 [12] and disrupts senescence by inhibiting p16INK4A and DNA damage pathways via suppression of CHD7 and TNRC6A [13]. TGF-β1 signaling is inhibited by miR-30c genes [42, 43].

This study showed that miR-30c-1 ameliorated TGF-β1-induced cell cycle cessation and TGF-β1-induced cellular hypertrophy. In cell cycle analysis, TGF-β1 caused G0/G1 cell cycle cessation, which is similar to hCECs *in vivo*. hCECs respond to wound healing by increasing cell size, where the TGF-β signaling pathway may play an important role. miR-30c-1 blocked TGFβR2, [12] which is critical for TGF-β signaling. YAP is a member of hippo signaling pathway, which controls cellular senescence [44]. Hippo-YAP pathway increases organ size through LATS-induced phosphorylation [45].

SASP factors including IL-6, TNF-α, and MIF were increased by TGF-β1 stimulus. SASP factors, which are mostly proinflammatory proteins, are released from senescent cells and activate the immune system and the EMT [46]. NF-κB participates in the modulation of immunological processes that stimulate the production of SASPs [47]. SMAD2 is a component of the TGF-β1 signaling pathway, and after activation by TGF-β1, enters the nucleus and activates the target gene promoting EMT [48]. miR-30c inhibits NF-κB signaling, thus inhibiting the SASP factors. Knock-down of NF-κB activity has been reported to reduce secretion of SASP factors [49].

TGF-β1 significantly stimulated the secretion of IGF-I and PDGF-BB [50–52]. IGF-1 is the inducer for senescence and is linked to organismal aging [53]. Up-regulated IGF-1 signaling maintains p21 level via the Ras-mitogen activated protein (MAP) kinase pathway, which contributes to reduce proliferation, to elevate cellular senescence, and to promote aging phenotypes [53]. IGF signaling is inhibited by miR-30c [42]. PDGF-BB binds to cell membrane tyrosine kinase receptor, promotes wound healing, cell proliferation and EMT [20]. The number of PDGF-BB receptors increases in senescent cell [54], although TGF-β signaling has been described to induce the production of PDGF-B [51].

### miR-30c-1 ameliorates TGF-β1-induced cell death of hCECs

This study showed that miR-30c-1 eliminated TGF-β1-induced depolarization of mitochondrial membrane potential. Levels of cleaved caspase-3, cleaved caspase-9, and cleaved PARP were increased by TGF-β1, and this effect was ameliorated by miR-30c-1 treatment. Mitochondrial membrane potential is a reliable measure of cell stress and apoptosis and is decreased during apoptosis [55]. The opening of mitochondrial permeability transition pores causes the loss of the mitochondrial membrane potential, release of cytochrome c, loss of ATP, and increased free radical formation [36, 56]. Caspase 3 is a performer in apoptosis because it coordinates the degradation of cellular structures [57]. Caspase 9 not only cleaves caspases-3, -6, and -7, which are the apoptosis executioners, but also participates in the regulation of autophagy and can facilitate autophagosome formation [58]. PARP is one of several substrates of caspases, and PARP cleavage inhibits the necrosis during apoptosis and guarantees the proper caspase-mediated cell death [59]. miR-30c maintained mitochondria membrane potential and reduced expression of apoptotic factors. Senescence is resistant to apoptosis induced by genotoxic stress [60]. However, excessive intracellular ROS causes DNA-damage and mitochondrial dysfunction, leading to cell death [61]. Although senescence is resistant to cell death, it is a process leading to cell death [61, 62].

Autophagy is a cellular program characterized by the degrading and recycling protein aggregates and damaged organelles [63]. Autophagy can mediate the transition to a senescent phenotype [64]. Inhibition of miR-30 encourages myocardial hypertrophy via exorbitant autophagy [65]. miR-30 family inhibits the BECN1 expression and autophagy [66]. This study showed that miR-30c-1 treatment eliminated TGF-β1-induced autophagy. TGF-β1 induces autophagy through the SMAD and JNK pathways [67]. The lysosome is a cellular center for signaling, metabolism, and quality control [68], where autophagy acts as a regulated pathway that digests cytoplasmic components and organelles [63]. LC3 is a central molecule in the autophagy pathway [69], and during autophagy, LC3-I (a cytosolic form of LC3) is changed to LC3-II (LC3-phosphatidylethanolamine conjugate), which is an autophagosomal marker [70]. The expression of cytochrome P450 3A4, which participates in the energy metabolism, is altered by miR-30c-1 [11]. miR-30c participates in regulation of autophagy through direct targeting BECN1 [71].

## CONCLUSIONS

In conclusion, miR-30c-1 promoted the proliferation of hCECs by ameliorating TGF-β1-induced senescence and reducing cell death of hCECs. Therefore, miR-30c-1 may be a valuable therapeutic target for regeneration of hCECs.

## MATERIALS AND METHODS

### Cell culture and transfection

This study was reviewed and approved by the institutional review board/ethics committee of Hallym University Kangnam Sacred Heart Hospital and was conducted in accordance with the Helsinki Declaration. Cells were cultured in accordance with previously published methods [72]. The corneas from six donors were used. hCECs were detached from Descemet’s membrane by trypsinizing for 10 min. The cells were cultured in 6-well plates applied with a fibronectin–collagen combination (FNC) coating mix (Athena Environmental Sciences, Inc., Baltimore, MD) and passaged at a ratio of 1:3 [72].

hCECs were transfected with human miR-30c-1 (5′-UGUAAACAUCCUACACUCUCAGC-3′; Bioneer corp., Daejeon, Korea) or mimic negative control (SMC-2002, Bioneer corp.; miR-control) using Lipofectamine™ RNAiMAX reagent (Invitrogen, Carlsbad, CA). Cells were treated for 48–72 h and then collected for the experiments. The expression of miR-30c-1 was confirmed by RT-qPCR at 48 h after transfection. In addition, hCECs were treated with and without TGF-β1 (10 ng/mL) and miR-30c-1 or miR-control for 72 h to evaluate the effect of hsa-miR-30c-1 on TGF-β1-treated cells.

### Cell viability and proliferation assay

Cells (1 × 10^4^) per well were seeded in a 96-well plate and treated with miRNA for 48–72 h. Cell viability was evaluated using a cell counting kit-8 (CCK-8; Dojindo, Kumamoto) [73]. After incubation with CCK-8 solution for 1–2 h, a Synergy HTX (BioTEK, Winooski, VT) multi-mode reader was used for evaluating cell viability by measuring optical density (OD) at 450 nm [73]. A bromodeoxyuridine (BrdU) incorporation assay kit (Roche Diagnostics, GmbH, Mannheim, Germany) was employed for evaluating cell proliferation rate according to manufacturer’s protocol [72].

### Cell cycle analysis

Muse cell analyzer (Merck Millipore, Burlington, MA) and propidium iodide (PI) staining was used for evaluating cell cycle analysis [74].

### Immunofluorescent staining

Immunofluorescent staining was performed as previously described [75]. Rabbit anti-human nuclear factor kappa-light-chain-enhancer of activated B cells (NF-κB) antibody (sc-372; Santa Cruz Biotechnology, Santa Cruz, CA) was applied as primary antibody and fluorescein isothiocyanate-conjugated goat anti-rabbit IgG antibody was applied as secondary antibody [75]. Nuclear counterstaining was conducted with Hoechst 33342 dye. The pictures were taken using fluorescence microscopy (DMi8; Leica).

### Enzyme-linked immunosorbent assay (ELISA)

The conditioned medium was obtained and stored at –70°C until used to evaluate the SASP secretion levels. The levels of interleukin-6 (IL-6), tumor necrosis factor-α (TNF-α), macrophage migration inhibitory factor (MIF), insulin-like growth factor 1 (IGF-1) and platelet-derived growth factor-BB (PDGF-BB) in the conditioned medium were measured using commercial human IL-6, TNF-α, MIF, IGF-1, and PDGF-BB ELISA kits (R&D Systems, Minneapolis, MN) [76]. In brief, capture anti-human IL-6, TNF-α, MIF, IGF-1, and PDGF-BB antibodies was applied to each well of 96-well plates overnight at 25°C. The wells were incubated with a blocking buffer containing 1% (w/v) BSA at 25°C for 1 h. Standard dilutions, 100 μL, of the commercial prepared human IL-6, TNF-α, MIF, IGF-1, and PDGF-BB, together with the experimental samples were applied to each well. After incubation at 25°C for 2 h, the plates were treated with goat anti-human IL-6, TNF-α, MIF, IGF-1, and PDGF-BB antibody conjugated to horseradish peroxidase (HRP) at 25°C for 2 h. Then, the plates were treated with a color reagent (3,3′,5,5′-tetramethylbenzidine [TMB]) for 20 min to obtain a blue color. Then, 1 M H_2_SO_4_ stop solution was applied. The OD was evaluated at 450 nm using a Synergy HTX multi-mode reader.

### Western blot

Radioimmunoprecipitation assay buffer (Biosesang, Seoul), supplemented with phosphatase (PhosSTOP; Roche, Basel) inhibitor cocktails and protease (Sigma-Aldrich, St. Louis, MO), was employed to obtain total proteins. SDS-PAGE electrophoresis and western blotting was conducted according to standard protocols. In brief, 5% skim milk was applied for inhibiting the nonspecific binding. The primary antibodies were as follows: rabbit anti- extracellular signal-regulated protein kinases 1 and 2 (ERK1/2) antibody (ab17942, Abcam); rabbit anti- phospho-ERK1/2 (pERK1/2) antibody (ab4819, Abcam); mouse anti- SMAD2/3 antibody (sc-133098, Santa Cruz); rabbit anti-p38 antibody (sc-535, Santa Cruz); mouse anti-p-p38 antibody (sc-7973, Santa Cruz); rabbit anti-p63 antibody (ab124762, Abcam); mouse anti-YAP antibody (sc-376830, Santa Cruz); rabbit anti-caspase 3 antibody (sc-7148, Santa Cruz); mouse anti-caspase 9 antibody (sc-56076, Santa Cruz); mouse anti-LC3 antibody (M186-3, MBL); rabbit anti-PARP antibody (sc-9542, Santa Cruz); or rabbit anti-GAPDH antibody (LF-PA0212, Abfrontier). After washing, an HRP-conjugated secondary antibody and a WEST-Queen™ western blot detection kit (iNtRON Biotechnology, Seongnam, Kyounggi-do) were applied for the detection of protein bands. Video image analysis (Luminograph II, Atto, Tokyo) was used to quantify the data [72].

### Real time reverse transcription polymerase chain reaction (RT-qPCR)

ReliaPrep™ RNA Miniprep Systems (Promega Cooperation, Madison, WI) was used to extract total RNA. Nanodrop method was used to measure RNA concentrations. GoScript™ Reverse Transcription System (Promega Cooperation) was used to synthesize first-strand cDNA from 0.2 μg of total RNA with oligonucleotide primers. AccuPower^®^ 2X GreenStar™ qPCR Master Mix (Bioneer) with RT-qPCR primer was used to perform RT-qPCR. The thermocycling parameters were as follows: 95°C for 10 minutes, 40 cycles at 95°C for 15 sec and at 60°C for 1 min [72]. SYBR green fluorescence intensity was taken. *β-actin* gene served as a refence gene. Melting curve analysis was employed to identify the purity of amplified products. ΔΔCq method was used to analyze RT-qPCR [72]. The reverse transcription primers were described in Supplementary Table 1.

### RNA sequencing analysis

ReliaPrep™ RNA Miniprep Systems (Promega Cooperation) was used for extraction of RNA from the cultured hCECs. RNA concentrations were measured by Nanodrop equipment. RNA sequencing analysis was conducted by Macrogen, lnc. (Seoul, http://www.macrogen.com/) [77]. In this study, gene expression values were obtained through transcriptome sequencing of Homo sapiens. NEBNext^®^ Ultra™ DNA Library Prep Kit for Illumina^®^ (NEB, USA) was used for libraries preparation of RNA samples. The sequencing libraries were prepared by random fragmentation of cDNA sample, which was followed by PCR amplification [77]. Illumina Hiseq 2500 platform was used for sequencing, which was performed by Macrogen, lnc [78, 79]. Fragments per kilobase of transcript per million mapped (TPM) value was employed to interpret individual gene expression level [77].

### Senescence-β-galactosidase assay

Senescence-β-galactosidase (SA-β-gal) staining kit (Biovision) was employed. Briefly, after the media was removed from the cells, each well was incubated in fixative solution for 10–15 min at 25°C. The SA-β-gal staining solution was applied at 37°C overnight in a dry incubator.

### Intracellular and mitochondrial oxidative stress evaluation

Dichloro-dihydro-fluorescein diacetate (DCFH-DA; Invitrogen, Carlsbad, CA) was employed to evaluate intracellular ROS levels. Cells were plated in cover glass-bottomed dishes and treated with miR-control or miR-30c-1. Cells were incubated with DCF-DA (10 μM) at 37°C for 1 h, washed, and observed under fluorescence microscopy (DMi8; Leica).

MitoSOX™ Red (Invitrogen) was employed to assess mitochondrial superoxide production. The cells were stained with MitoSOX™ reagent (5 μM) for 10 min at 37°C [74]. Muse cell analyzer was used to evaluate fluorescence intensity.

### MitoTracker red and lysosome staining

Mitochondrial mass was measured using MitoTracker red FM fluorescent probe (Invitrogen). Cells (1 × 10^4^) were treated with 200 nM MitoTracker red FM fluorescent probe for 30 min. Muse cell analyzer was used for analysis [72]. The cells were seeded in cover glass-bottom dishes, incubated with LysoTracker green (Invitrogen; 50 nM) and MitoTracker red (200 nM) for 30 min. The cells were observed under fluorescence microscopy (DMi8; Leica).

### MitoPotential assay

Mitochondrial membrane potential was assessed using Muse™ MitoPotential assay (Merck Millipore) [73]. Muse™ Cell Analyzer was used for analysis.

### Statistics

Data were presented as mean ± standard deviation. Experiments were repeated three times, and a representative experiment is shown. To compare two groups, an independent *t*-test was applied. GraphPad Prism 8 was used for statistical analyses.

## Supplementary Material

Supplementary Materials

Supplementary Figures

Supplementary Table 1
