# Supplementary material for: miR-30c-1 encourages human corneal endothelial cells to regenerate through ameliorating senescence
Source: Aging (Albany NY). 2021 Mar 19;13(7):9348–72. doi: 10.18632/aging.202719 (PMC8064150; doi:10.18632/aging.202719)
Supplement: Supplementary Materials [file aging-13-202719-s001.pdf]

SUPPLEMENTARY MATERIALS

miR-30c-1 inhibitor induces the senescence of hCECs

The number of SA-β-gal positive cells increased in treated with miR-30c-1-3p inhibitor or miR-30c-1-5p inhibitor ($p < 0.001$ for all; Supplementary Figure 1A–1B). Intracellular oxidative stress levels were elevated in treated with miR-30c-1-3p inhibitor or miR-30c-1-5p inhibitor ($p < 0.001$ for all; Supplementary Figure 1C–1D). Cell viability decreased in treated with miR-30c-1-3p inhibitor or miR-30c-1-5p inhibitor ($p = 0.004$ and <0.001; Supplementary Figure 1E). BrdU proliferation rate decreased in treated with miR-30c-1-3p inhibitor or miR-30c-1-5p inhibitor ($p = 0.001$ and <0.001; Supplementary Figure 1F)

TGF-β1 induces the senescence of hCECs

BrdU proliferation rate decreased at 24 h, 48 h, and 72 h compared with 0 h after TGF-β1 treatment ($p = 0.026$, $p = 0.047$, and $p = 0.023$, respectively; Supplementary Figure 2A). Representative images of cell cycle analysis are shown in Supplementary Figure 2B. Cell cycle analysis showed that the number of cells in G0/G1 phase increased at 48 h and 72 h compared with 0 h ($p < 0.001$ for both; Supplementary Figure 2C), while the number of cells in S phase decreased at 48 h and 72 h compared with 0 h ($p = 0.005$ and $p = 0.003$, respectively; Supplementary Figure 2D), and the number of cells in G2/M phase decreased at 48 h and 72 h compared with 0 h ($p = 0.006$ and $p = 0.010$, respectively; Supplementary Figure 2E). The number of SA-β-gal positive cells increased at 24 h, 48 h, and 72 h compared with 0 h after TGF-β1 treatment ($p < 0.001$, $p < 0.001$, and $p < 0.001$, respectively; Supplementary Figure 2F–2G). Cell size increased at 48 h and 72 h compared with 0 h ($p = 0.005$ and $p < 0.001$, respectively; Supplementary Figure 2H–2I).

Intracellular oxidative stress levels were elevated by TGF-β1 at 48 h and 72 h compared with 0 h ($p = 0.034$ and $p = 0.001$, respectively; Supplementary Figure 3A–3B). The pERK1/2 level increased over time after TGF-β1 treatment (Supplementary Figure 3C). The percentage of cells with depolarized mitochondrial membrane potential was elevated at 48 h and 72 h compared with 0 h ($p = 0.002$ and $p = 0.001$, respectively; Supplementary Figure 3D–3E).
