# Supplementary material for: miR-30c-1 encourages human corneal endothelial cells to regenerate through ameliorating senescence
Source: Aging (Albany NY). 2021 Mar 19;13(7):9348–72. doi: 10.18632/aging.202719 (PMC8064150; doi:10.18632/aging.202719)
Supplement: Supplementary Figures [file aging-13-202719-s002.pdf]

SUPPLEMENTARY FIGURES

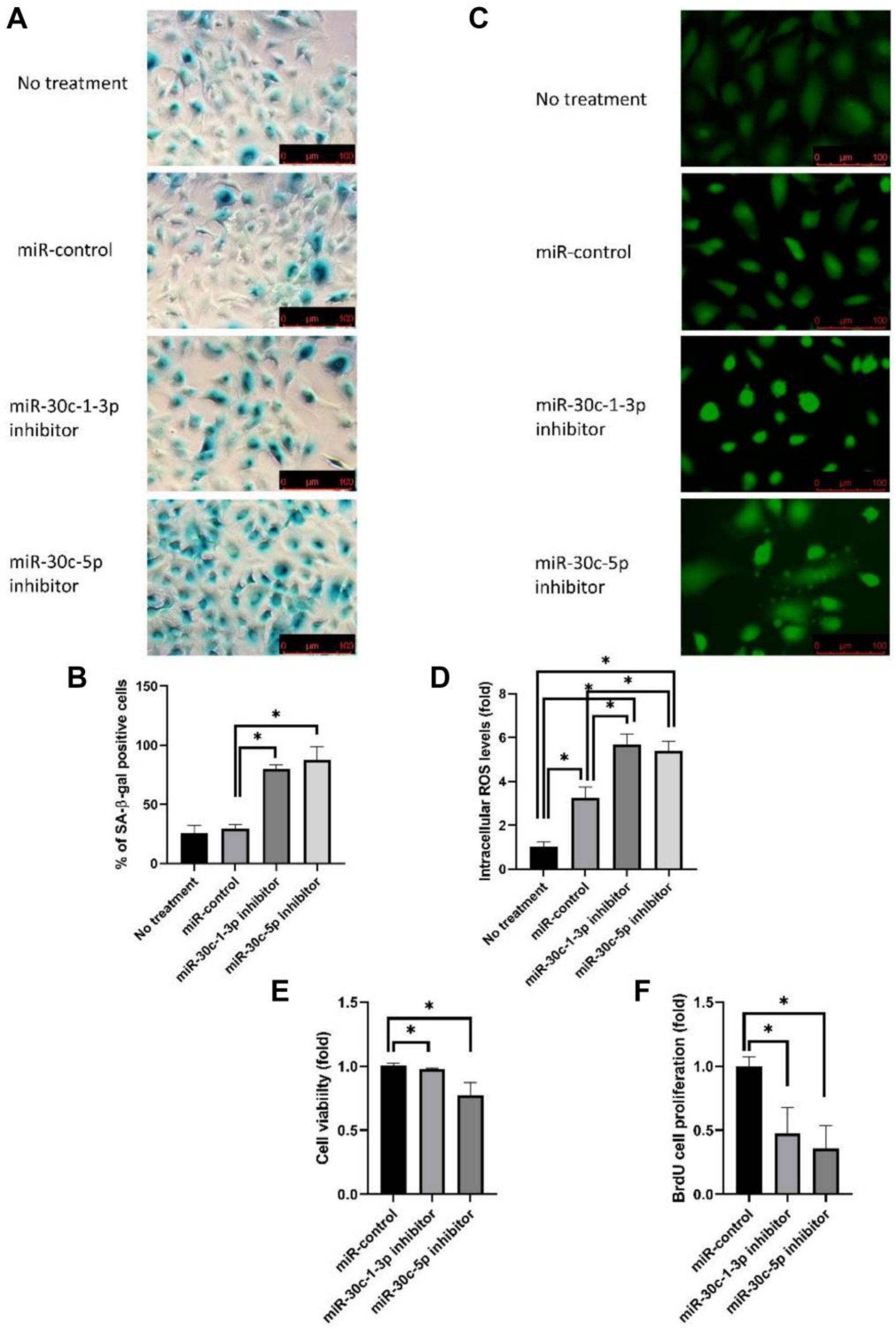

Supplementary Figure 1. miR-30c-1 inhibitor induces the senescence of hCECs. (**A**–**B**) Senescence-associated β-galactosidase positive cells were less shown in treatment with miR-30c-1 inhibitors. (**C**–**D**) Intracellular oxidative stress levels were increased in treatment with miR-30c-1 inhibitors. (**E**–**F**) Cell viability and BrdU cell proliferation rate were decreased in treatment with miR-30c-1 inhibitors. *statistically significant.

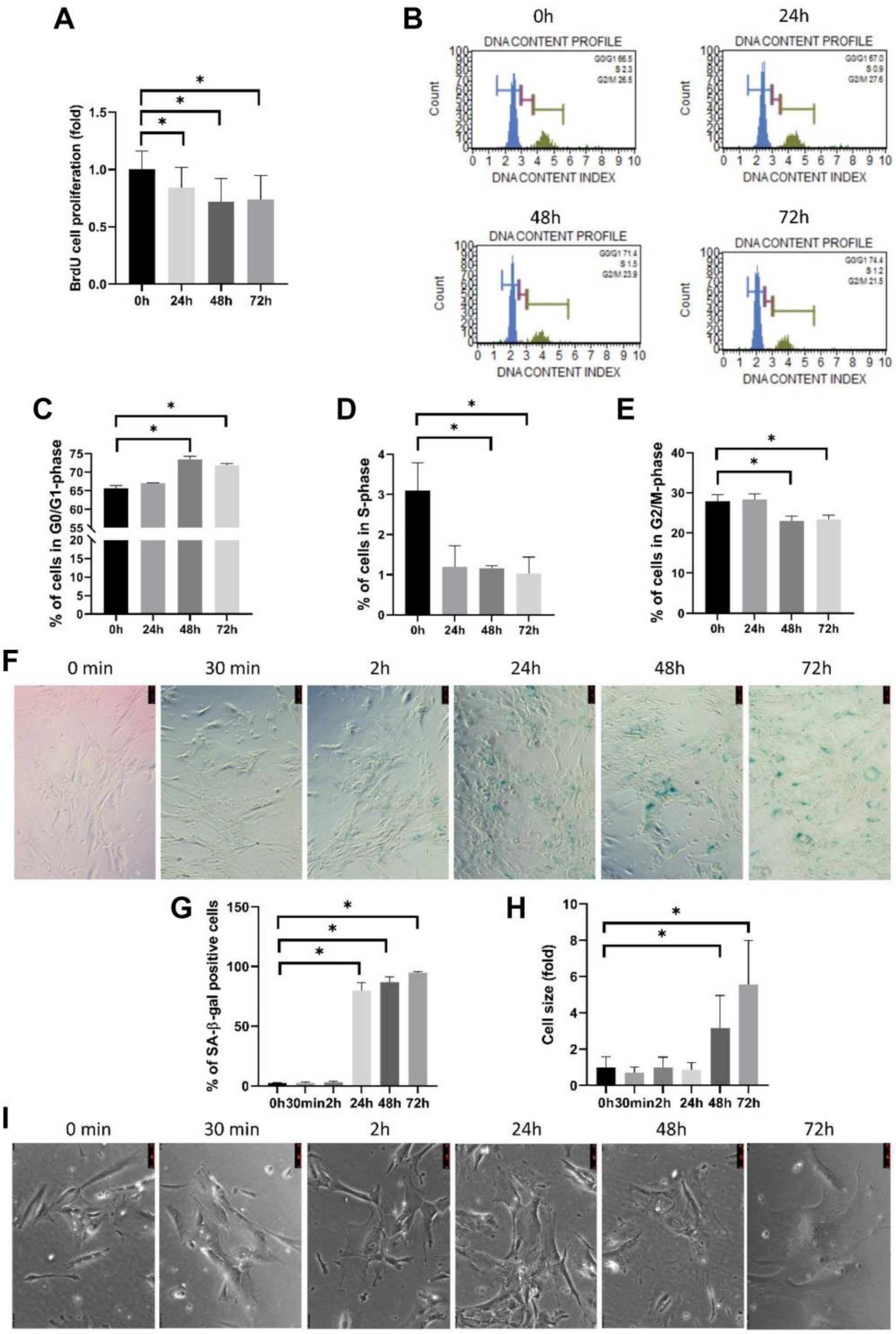

Supplementary Figure 2. Effect of TGF-β1 on cell cycle arrest and senescence. (**A**) Cell proliferation was measured using BrdU proliferation assay. (**B**) Cell cycle analysis using by DNA content measurement. (**C**–**E**) The percentages of cells in G0/G1-phase (C), in S-phase (D) and G2/M-phase (E) were analyzed. (**F**) Representative images of senescence-β-galactosidase (SA-β-gal) staining. (**G**) The percentage of SA-β-gal positive cells was quantified. (**H**) Representative images of cell shape. (**I**) Cell size increased over time after TGF-β1 treatment. *statistically significant.

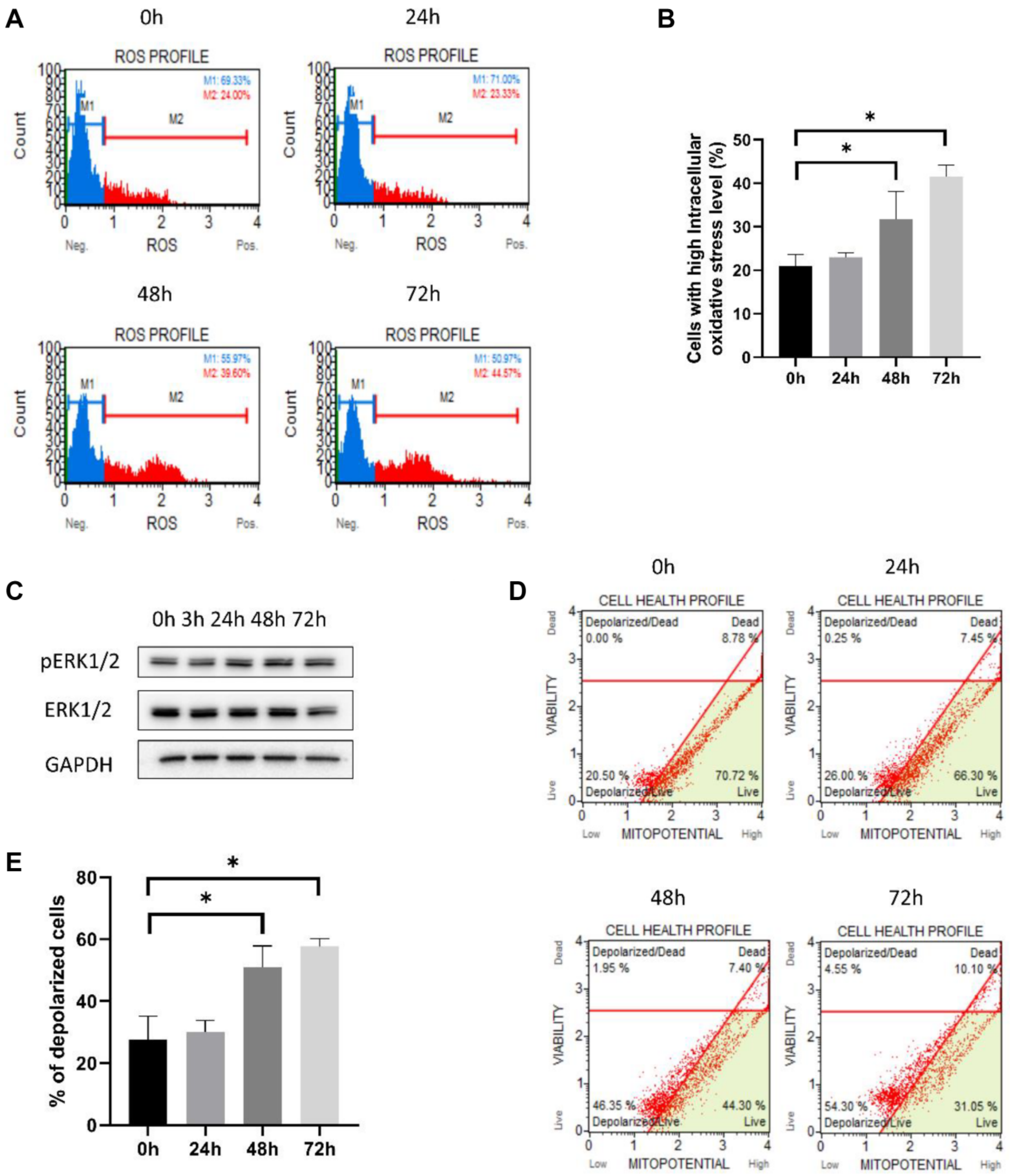

Supplementary Figure 3. Effect of TGF-β1 on mitochondria after TGF-β1 treatment. (**A**) Representative images of oxidative stress levels. (**B**) The percentage of cells with high intracellular oxidative stress level increased over time. (**C**) Activation of ERK increased over time. (**D**) Representative images of mitochondrial membrane potential. (**E**) The percentage of depolarized cells increased over time. *statistically significant.
