# Supplementary material for: miR-30c-1 encourages human corneal endothelial cells to regenerate through ameliorating senescence
Source: Aging (Albany NY). 2021 Mar 19;13(7):9348–72. doi: 10.18632/aging.202719 (PMC8064150; doi:10.18632/aging.202719)
Supplement: Supplementary Table 1 [file aging-13-202719-s003.pdf]

SUPPLEMENTARY TABLE

Supplementary Table 1. Primers for RT-qPCR.

Gene	Forward primer sequence	Reverse primer sequence
miR-30c-1	TGTGTAAACATCCTACACTCTCAG	GAGTAAACAACCCTCTCCCA
